# Development of a safe drug administration assessment instrument for nursing students*

**DOI:** 10.1590/1518-8345.2989.3246

**Published:** 2020-02-03

**Authors:** Karen Domínguez Cancino, Marisol Arias, Erika Caballero, Eliana Escudero

**Affiliations:** 1 Universidad Finis Terrae, Escuela de Enfermería, Santiago, Chile.; 2 Universidad María Auxiliadora, Facultad de Salud, Peru.; 3 Universidad Autónoma de Chile, Chile.

**Keywords:** Nursing Research, Medication Errors, Education, Nursing, Patient Safety, Simulation Training, Guideline Adherence, Pesquisa em Enfermagem, Erros de Medicação, Educação em Enfermagem, Segurança do Paciente, Treinamento por Simulação, Fidelidade a Diretrizes, Investigación en Enfermería, Errores de Medicación, Educación en Enfermería, Seguridad del Paciente, Entrenamiento Simulado, Adhesión a Directriz

## Abstract

**Objective:**

to determine the content and face validity of a safe drug administration assessment instrument for nursing students.

**Method:**

quantitative, descriptive study. The literature on medication errors made by students was analyzed, and an instrument was developed using the Architecture of Integrated Information Systems and the Work Breakdown Structure. Face validity was analyzed using the nominal technique, with experts in education, management, research and/or clinical practice.

**Results:**

nine experts participated in the validation process, which resulted in an instrument containing 8 sub-processes and 58 items, adjusted to the simulation environment and to clinical practice.

**Conclusion:**

the instrument may be used for the evaluation of safe drug administration by nursing students, especially in a simulation environment.

## Abstract (pt)

**Objetivo:**

determinar a validade de face e conteúdo de um roteiro de avaliação de administração segura de medicamentos em estudantes de enfermagem.

**Método:**

estudo quantitativo, descritivo. Analisou-se a literatura relativa a erros de medicação em estudantes e elaborou-se um roteiro utilizando a metodologia Arquitetura Integrada de Sistema de Informação e a Estrutura de Decomposição do Trabalho. Analisou-se a validade de face utilizando a técnica nominal com especialistas em educação, gestão, pesquisa e/ou prática clínica.

**Resultados:**

nove especialistas participaram de todo o processo de validação, do qual se obteve um roteiro com 8 subprocessos e 58 itens, ajustados ao ambiente de simulação e prática clínica.

**Conclusão:**

o roteiro se encontra em condições de ser testado na avaliação da administração segura de medicamentos em estudantes de enfermagem, principalmente em ambiente de simulação.

## Abstract (es)

**Objetivo:**

determinar la validez de aspecto y contenido de una pauta de evaluación de administración segura de medicamentos en estudiantes de enfermería.

**Método:**

estudio cuantitativo, descriptivo. Se analizó la literatura respecto a errores de medicación en estudiantes y se creó una pauta utilizando la metodología Arquitectura Integrada de Sistema de Información y la Estructura de Descomposición del Trabajo. Se analizó la validez de aspecto utilizando la técnica nominal con personas expertas en educación, gestión, investigación y/o práctica clínica.

**Resultados:**

Nueve personas expertas participaron durante todo el proceso de validación, se obtuvo una pauta con 8 subprocesos y 58 ítems, ajustados al ambiente de simulación y práctica clínica.

**Conclusión:**

La pauta se encuentra en condiciones de ser probada en la evaluación de la administración segura de medicamentos en estudiantes de enfermería, especialmente en el ambiente de simulación.

## Introduction

Although 18 years have passed since the publication of the Institute of Medicine’s (IOM) “To Error is Human”^(^ 1 ^)^, preventable adverse events remain an unresolved issue, and are a cause of major concern today^(^ 2 ^)^. Adverse events may reach values close to 10%, with greater prevalence of those related to drug use, anesthesia and surgery, and hospital infections^(^ 3 ^)^. Drug use errors are associated with somewhere between 3 and 6% of hospital admissions, 30 to 40% of which are the result of avoidable errors^(^ 4 ^)^. A study in Chilean hospitals found a medical error rate of 1.2 x 1,000, which is considered high. These errors occurred in medical services and Critical Patient Units, the most frequent being related to drug administration (47%) and dispensation (27%)^(^ 5 ^)^.

Prevention strategies and the development of an event notification culture are the pillars to avoid such errors^(^ 6 ^)^. Recognizing the causes of drug administration errors and implementing safety strategies before, during and after this process results in a significant increase in the quality of patient care^(^ 6 ^)^.

Different studies have shown that nursing professionals play a crucial role in preventing medication errors in clinical practice^(^ 7 ^-^ 8 ^)^, as these correspond to the last barrier separating the patient from medication. Despite the acknowledgment of this fact, professionals are not properly trained in how to deal with errors, especially due to the relationship of these events with feelings of guilt, shame and limited scientific knowledge, as well as the fear related to ethical and legal responsibilities^(^ 7 ^)^. Given the responsibility of nursing professionals and the high prevalence of medication errors in clinical settings, especially those administered parenterally^(^ 9 ^)^, the Finis Terrae University’s School of Nursing, as evidence of its high commitment to patient safety, used an information management system with care management indicators to develop a drug administration protocol^(^ 10 ^-^ 11 ^)^ based on the available information on the subject^(^ 1 ^,^ 3 ^-^ 4 ^,^ 7 ^-^ 9 ^,^ 12 ^-^ 13 ^)^, applying the Architecture of Integrated Information Systems (ARIS)^(^ 14 ^)^ and the Work Breakdown Structure (WBS)^(^ 15 ^)^. The purpose of the protocol is incorporating the processes of drug management, administration and dispensation, with their associated records, as a fundamental pillar in the training of students, emphasizing the concepts of safety and quality of health care. The protocol establishes 9 sub-processes, including safety breaks, that favor the safe administration of drugs: reassessment of prescription, evaluation, dispensation, transcription, preparation, administration, error reporting, follow-up and monitoring of therapeutic, side and adverse effects, and provision of information to the patient. This protocol has been applied throughout the course since 2016.

At the international level, it is recognized that there are at least three steps in the drug administration process^(^ 14 ^-^ 15 ^)^, varying between different countries, mainly due to legal conditions and the implementation of computerized processes, interventions aimed at making this process safer^(^ 16 ^)^. In countries such as Chile, the nurse is responsible and involved in all stages of the process, either by performing, delegating or supervising these instances.

The aim of the present study was determining the face and content validity of a safe drug administration assessment instrument for nursing students.

## Method

This was a quantitative, descriptive study, conducted at the School of Nursing under the project “Assessment of the impact of the safe drug administration protocol for nursing students one year after its implementation”, with funding from an Annual Research Bidding Process (CAI) held by the Board of Research and Graduate Studies at the Finis Terrae University in 2017.

The study was carried out in two different phases, comprising the creation and validation of the instrument. In the creation phase, the safe drug administration protocol used in the School of Nursing was considered as base, consisting of nine sub-processes, each with a definition, a responsible party, aspects to verify, observations, records and safety breaks to prevent possible risk situations. For the instrument’s creation, the indications for each sub-process were transformed into observable actions expected in the process of safe parenteral drug administration, performed in the simulation environment or in clinical practice, having as possible answers “yes”, “no” and “not applicable”. This process was carried out by one of the researchers and then revised and discussed with the others.

The second phase consisted in the instrument’s face validation by the experts using the nominal group technique^(^ 17 ^)^. Nationally prominent people in the fields of research, management, medical education and clinical simulation were contacted, as well as clinical professionals, both pediatric and adult, who worked in education and health institutions, both public and private, and who had conducted present or past teaching activities at the school, being thus familiar with the Institution’s curriculum^(^ 18 ^)^, totaling 17 experts.

An email containing an invitation letter was sent, along with a personal data survey, the safe drug administration protocol and the safe drug administration assessment instrument. Once the expert agreed to participate, he/she was asked to submit the personal data survey, containing information about his/her experience, graduate studies and publications in the above areas, also including an item concerning his/her coefficient of expertise^(^ 19 ^-^ 20 ^)^, used to decide whether the expert was qualified to carry out the process.

As indicated by the nominal technique, there was a remote and an in-person phase. In the remote phase, the experts were requested to send their assessment of the specific aspects, relevance and quality of the item. Relevance refers to the importance of including the item to measure the “safe drug administration” process, while quality refers to proper wording, and to whether the item is easy to understand and apply in different scenarios. A Likert-type scale was used to determine the level of agreement with each item, considering the categories “strongly disagree”, “disagree”, “neither agree nor disagree”, “agree” and “strongly agree”. In addition, a space for comments was provided at the end of each sub-process included in the instrument.

Once the information had been obtained, the data were revised by analyzing the experts’ evaluations and agreement level, as well as the comments regarding each item, after which they were invited to attend an in-person session to unify the criteria.

For the development of the in-person session, a presentation was prepared in audiovisual media to allow discussing the relevance of the item’s inclusion and the clarity with which it was exposed, in the case of aspects with at least one score in the categories “neither agree nor disagree”, “disagree” and “strongly disagree”. The items with scores in the categories “strongly agree” or “agree” were not considered, as there was no discrepancy in the analysis. The sessions were recorded and then transcribed verbatim for later analysis.

Subsequently, the instrument was applied by the evaluators, after the faculty had been trained in its application as well as that of the protocol, in a simulation environment built for a small group of students, two from the third year and two from the fourth year, with at least one year of experience with the protocol’s application, to analyze the instrument’s behavior in relation to the evaluation process commonly used at the school in high-stake instances taking place throughout the students’ career^(^ 18 ^)^. The objective was to test both regarding the realism of the clinical simulation scenario.

A low-fidelity simulation environment was built to test the instrument. Low-fidelity simulations correspond to a training activity wherein the professor guides and corrects the student in relation to a given technique^(^ 21 ^)^. The student is given prior instructions on how to carry out the learning experience (briefing), as well as ongoing feedback, allowing him/her to correct and improve his/her performance. The simulation lasted 10 minutes, and the evaluators observed it from behind a mirrored glass. The student was asked to prepare and administer a parenteral drug to control the patient’s pain, after which he/she had 5 minutes to register the procedures performed. Each student was evaluated by two experts, who could ask the researcher general questions without showing their results to someone else so as not to influence other people’s answers. In addition, they could suggest changes to the instrument based on what was observed in the scenario and on the ease of its application.

Regarding the ethical aspects, the four bioethical principles (non-maleficence, beneficence, justice and autonomy) and the criteria of Ezekiel Emanuel^(^ 22 ^)^ were considered, in addition to the norms and laws applicable to research with human beings in the country. The experts signed an invitation letter, agreeing to participate in the research and ensuring the confidentiality of the research data. Additionally, the students who participated in the application of the instrument were asked to sign an informed consent form. The study was approved by the research ethics committee of the Finis Terrae University (Resolution No. 15/2017).

## Results

The creation/validation of a standard observation instrument followed the analysis of the initial one by the researchers. An instrument with 9 sub-processes containing 63 items answered as either “yes”, “no” and “not applicable” was obtained, each with a column in which the expert and/or evaluator could comment on the students’ performance.

Of the 17 experts contacted, 13 participated in the remote stage, and 9 attended the discussion session in person or via videoconference.

Characteristics of the experts: of the 13 participants, 92% were women, aged from 34 to 61 years old; 92% were nursing professionals, and 100% had graduate degrees, 40% had undergraduate degrees, and 85% had master’s degrees. Most specialists were active in more than one area (research, management, medical education and clinical simulation). The average time of experience with safe drug administration was 8 years (values between 0 and 27 years).

Table 1 presents the coefficient of expertise globally. Of the experts, 92% actually met the criteria in terms of knowledge level and sources of arguments for validating the instrument. Those with expertise categorized as low were excluded.

**Table 1:** Coefficient of expertise (N=13). Santiago, Chile, 2017

	Mean	Minimum	Maximum	Number (%)
Knowledge level	0,72	0,3	0,9	
Sources of arguments	0,85	0,5	0,9	
Final degree of expertise	0,79	0,45	0,95	
*Category of the coefficient of expertise*				
Low				1 (8,0%)
Medium				6 (46%)
High				6 (46%)

Discussion sessions: two sessions were held on different days of March, lasting 2 hours and 30 minutes. Using the presentation in audiovisual media, the answers of the experts were analyzed, focusing on the items with scores in the categories “do not agree or disagree”, “disagree” and “strongly disagree”, in relation to relevance and quality. During the discussion, the complexities of the process were addressed, along with the experiences of each of the participants. In addition, the wording required to make the instrument more comprehensible was analyzed, and some steps were limited due to the difficulty of measuring certain aspects in a single observation. It was decided to merge, limit or simplify some sub-processes and items, and the wording was adapted to actions deemed as easily observable in evaluation environments. In this phase, an instrument containing 8 sub-processes and 61 evaluation items was obtained.

After the instrument’s application in the simulation environment, the evaluators suggested the removal of 3 items, as well as minor changes to the wording used.

The final instrument consisted of 8 sub-processes: reassessment of prescription, evaluation, dispensation, transcription, preparation, administration, error reporting, follow-up and monitoring, and provision of information to the patient, evaluated in 58 items. The sub-processes, items, and safety breaks are shown in Figure 1.

**Figure 1:** Configuration of the final instrument. Santiago, Chile, 2017

Sub-processes	Quantity of items and safety breaks
Reassessment of drug prescription	10 items, 1 safety break
Evaluation	1 item
Transcription to the medical records (if applicable)	6 items
Drug Preparation	18 items, 1 safety break
Administration of the medication	14 items, 1 safety break
Error reporting (if applicable)	4 items
Follow-up and monitoring of the patient	1 item
Provision of information about the treatment to the patient	2 items

## Discussion

This research aimed to determine the face and content validity of a safe drug administration assessment instrument for nursing students. An instrument with 8 sub-processes was developed, including: reassessment of prescription, evaluation, dispensation, transcription, preparation, administration, error reporting, follow-up and monitoring of therapeutic, side and adverse effects, and provision of information to the patient, evaluated in 58 items, having been considered by experts and evaluators as easy to apply and transferable to clinical practice.

The use of judgment by experts as a strategy to validate instruments in the nursing field, especially processes involving observable actions, has been observed^(^ 23 ^-^ 27 ^)^. The most frequently used technique is the Delphi method, which allows blindly obtaining information from experts in three rounds in order to estimate the final percentage of agreement^(^ 23 ^-^ 27 ^)^. The participation of experts located in areas that are geographically more distant and the reduction of consensus-derived problems are seen as competitive advantages compared to the nominal group technique^(^ 28 ^)^. However, the first point was relatively remedied in this research, as the experts were allowed to participate in the different sessions via videoconferences. On the other hand, the assessment instrument was applied in a simulated environment, following the usual academic practices performed in our school, without considering a process for testing its reliability^(^ 23 ^-^ 27 ^)^.

Research shows that some of the reasons why safe drug administration protocols fail or have poor adherence are, on the one hand, related to poorly learned and highly ingrained professional practices^(^ 9 ^)^, and, on the other, due to the limited opportunities to train and acquire competence in this practice^(^ 29 ^-^ 30 ^)^. This justifies the implementation of instruments such as this in academic processes. Studies have associated simulation focused on skills with the development of safe health care practices^(^ 31 ^-^ 32 ^)^. Review conducted in 2017 highlights the importance of training with simulation experiments^(^ 33 ^)^. This review shows the improvements achieved by subjecting nursing professionals and students to learning experiences using this methodology, especially in relation to safe drug administration. Similarly, evidence from 2019 shows improvements in the results of the training of nursing professionals subjected to simulations, with promotion of very specific skills such as those associated with assessment and emergency^(^ 34 ^)^, supporting the performance of simulation-based interventions focused on different critical areas related to medication errors in the early stages of the professionals’ training^(^ 35 ^)^.

The strengths of the study include it being the first published initiative to standardize the drug administration process by identifying specific steps required to prevent nursing students from making medication errors. The instrument allows the assessment of competency and provision of standardized feedback. It was developed as a standardized technique to be used in the professionals’ training, with the aim of ensuring the quality and safety of the process, both in simulation activities and clinical practice^(^ 36 ^-^ 37 ^)^. Training students in safety practices may lead to changes in the way things are done, as well as decrease the rate of adverse events in the future. Training and practice in clinical simulation favor the progressive and repetitive application of sub-processes until the achievement of a certain skill, also helping students reflect on and correct errors^(^ 38 ^)^, benefits which have been tested for the teaching of safe drug administration^(^ 39 ^)^. Studies show that students and professionals value the use of nontraditional strategies such as clinical simulation in the teaching and learning of certain habitual practices, like drug administration^(^ 40 ^)^.

One of the weaknesses of the study is the fact that the instrument has no criterion and construct validity. This is related to the lack of other instruments to measure the process (concomitant) and of hard data on medication errors made by students (predictive), which poses a new challenge for the team to conduct further research and continue improving it.

On the other hand, during the validation process, it was difficult to obtain a consensus from the experts, due to the heterogeneity of opinions and lack of Chilean regulations on drug administration, which is line with what has been observed internationally.

Another difficulty that could be solved, but is still worth mentioning, is that there was a certain heterogeneity in the application of the protocol and assessment instrument in the simulated scenario, although the professors had been trained in it. This is partly due to the fact that the professors’ training does not necessarily lead to competence in the performance of the simulation methodology^(^ 41 ^)^.

Studies such as this will enable undergraduate students to acquire skills in safe healthcare practices. The nursing faculty has the responsibility to generate strategies to reduce adverse events at the end of the process.

## Conclusion

The instrument is in conditions of being used by the nursing faculty to evaluate the safe administration of medication by nursing students, especially in a simulation environment.
